# Clinical applications of artificial intelligence-driven nitric oxide: a bibliometric and scientific mapping analysis

**DOI:** 10.4103/mgr.MEDGASRES-D-25-00096

**Published:** 2026-01-06

**Authors:** Zhen Liu, Ainikaer Abulaiti, Yan Zhao, Junting Zang, Guohua Li, Li Shu, Paerhati Wahafu, Maihemuti Yakufu

**Affiliations:** 1 Orthopedic Research Center, Sixth Affiliated Hospital of Xinjiang Medical University, Urumqi, Xinjiang Uygur Autonomous Region, China; 2 Department of Orthopedic Surgery, Orthopedic Center, The First Hospital of Jilin University, Changchun, Jilin Province, China

**Keywords:** artificial intelligence, bibliometrics, cardiovascular disease, Citespace, deep learning, knowledge graph, machine learning, nitric oxide, VOSviewer, Web of Science

## Abstract

Nitric oxide, a pivotal endogenous signaling molecule, plays crucial roles in cardiovascular regulation, immune response, and neuromodulation. The rapid advancement of artificial intelligence technologies offers novel approaches to optimize real-time nitric oxide monitoring, dosing regimens, and toxicity prediction. Current interdisciplinary research on the artificial intelligence-driven nitric oxide intersection remains fragmented, with a lack of systematic investigations into knowledge architecture, technological evolution, and translational barriers. This study addressed this critical gap by presenting the knowledge graph-based analysis of artificial intelligence-driven nitric oxide system. A total of 384 relevant articles (2005–2024) were retrieved in the Web of Science Core Collection and analyzed using CiteSpace, VOSviewer, and Bibliometrix R package. Annual publications demonstrated a biphasic growth, accelerating after 2017 in tandem with breakthroughs in artificial intelligence architectures. Although China and the United States were dominated in this field, international collaborations exhibited a core-periphery structure. Research themes predominantly focused on cardiovascular and respiratory diseases, with underdeveloped applications in neuroimmunology and infectious diseases. Highly cited literature that emphasized photodynamic therapy and disease risk assessment revealed insufficient integration between artificial intelligence algorithms and fundamental nitric oxide mechanisms. Keyword evolution analysis identified a paradigm shift from traditional mechanisms (e.g., “blood pressure,” “inflammation”) to technology-driven approaches (e.g., “machine learning, ” “deep learning”). Clinical translation has faced challenges, including data heterogeneity, algorithm interpretability, and deficiencies in multicenter validation. This pioneering study systematically delineates the knowledge framework and translational bottlenecks in artificial intelligence-driven nitric oxide convergence. Future research should prioritize artificial intelligence modeling of nitric oxide dynamic metabolism, the development of explainable algorithms, and prospective clinical trials to bridge the laboratory-to-clinic gap.

## Graphical abstract

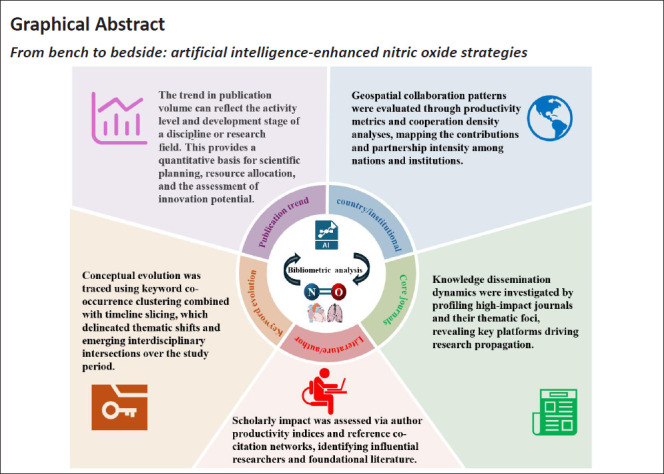

## Introduction

Nitric oxide (NO) is a pivotal endogenous signaling molecule that plays critical roles in cardiovascular relaxation, immune regulation, and neural transmission.^1^ Its medical applications have transitioned from fundamental research to clinical therapeutics. Examples include inhaled NO therapy for neonatal persistent pulmonary hypertension,^2^,^3^,^4^ acute respiratory distress syndrome,^5^,^6^ and acute respiratory failure.^7^ Additionally, NO donor drugs (e.g., nitroglycerin) serve as first-line treatments for cardiovascular diseases,^8^ while emerging technologies including NO-releasing agents,^9^ fractional exhaled NO (FeNO) monitoring,^10^,^11^ and NO synthase (NOS)-targeted therapies^12^ are being progressively integrated into clinical practice. However, the clinical implementation of NO faces substantial challenges due to its concentration- and spatiotemporal-dependent biological effects, narrow therapeutic window, and unpredictable toxicity risks.

The rapid advancement of artificial intelligence (AI) in healthcare has created unprecedented opportunities for disease diagnosis, therapeutic decision-making, personalized medicine, and biological mechanism elucidation.^13^ Cutting-edge AI technologies—particularly deep learning, machine learning, natural language processing, and computer vision—have significantly enhanced the learning efficiency and predictive capabilities of medical models. These innovations have demonstrated clinical utility across multiple disciplines, including internal medicine^14^ and radiology^15^ with certain AI/machine learning models outperforming human evaluators in specific diagnostic tasks.^16^ Notably, AI-driven approaches offer transformative potential for overcoming NO-related clinical limitations through machine learning-based analysis of dynamic NO metabolic networks, deep learning-optimized dosing regimens, and intelligent monitoring systems for real-time toxicity prediction.

Despite these advancements, current research on the NO-AI intersection exhibits a fragmented nature. Although isolated technical breakthroughs (e.g., convolutional neural network-based FeNO detection^17^) are frequently reported, systematic investigations into the field’s knowledge architecture, technological evolution, and interdisciplinary collaboration patterns remain absent. Existing bibliometric analyses predominantly focus on single disciplines (e.g., pharmacology or computer science), with a lack of holistic insights into the cross-disciplinary convergence of NO-AI. Critical barriers to clinical translation—including data heterogeneity, model interpretability, and validation protocol standardization—have been insufficiently addressed, obscuring emerging trends and research priorities.

To address these gaps, this study conducted a comprehensive bibliometric knowledge mapping analysis to rigorously examine research trends in NO-AI integration from 2005 to 2024. The primary objectives guide our research: (1) constructing knowledge maps of NO-AI convergence using CiteSpace, VOSviewer, and Bibliometrix R package; (2) identifying core journals, influential authors, and institutional collaboration networks; and (3) detecting research hotspots and forecasting future directions through evolutionary trajectory analysis.

## Methods

### Study design

**Figure 1:**
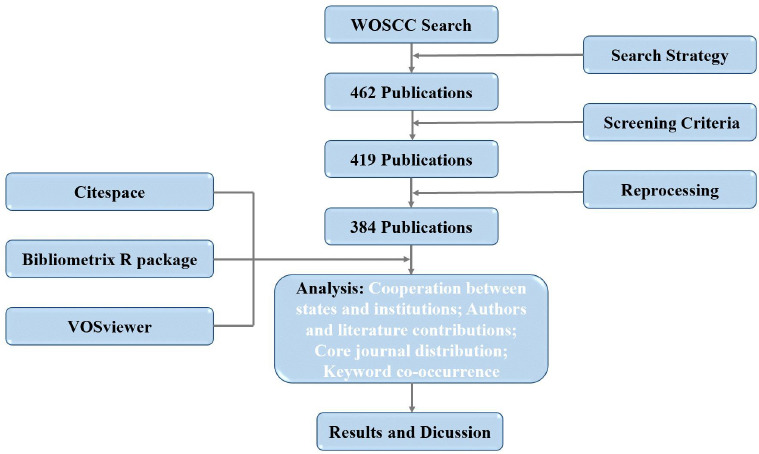
Literature screening flowchart for the NO-AI field. AI: Artificial intelligence; NO: nitric oxide; WoSCC: Web of Science Core Collection.

This investigation used bibliometric analysis to quantitatively evaluate research status and trends in the integration of NO and AI from 2005 to 2024. The research workflow consists of three main phases (**Figure 1**).

#### Data collection

The Web of Science Core Collection was the exclusive data source. We developed a systematic search algorithm that combined NO- and AI-related terms to retrieve core publications, with predefined inclusion/exclusion criteria ensuring dataset comprehensiveness and reliability.

#### Analytical tools

Knowledge mapping was performed using CiteSpace (version 6.4.1; Drexel University, Philadelphia, PA, USA) and VOSviewer (version 1.6.20; Leiden University, Leiden, Netherlands) to visualize research collaboration networks and thematic clusters. Complementary statistical analysis and visualization were conducted using the Bibliometrix R package (version v4.1.2; University of Naples Federico II, Naples, Italy). Trend fitting was performed using a power-exponential model implemented in Microsoft Excel 2021 (Microsoft Corporation, Redmond, WA, USA).

#### Data analysis

Following deduplication and preprocessing, we systematically examined: journal distribution patterns, author/institutional collaboration networks, country contribution patterns and keyword co-occurrence clusters. This multilevel analysis framework elucidates evolving research priorities and developmental trajectories.

### Data collection and processing

#### Search strategy

A three-tiered search strategy was developed in the Web of Science Core Collection to retrieve publications integrating NO and AI: (1) NO-related terms encompassed chemical nomenclature, functional descriptors, detection techniques, and molecular mechanisms; (2) AI-related terms covered generic algorithms (e.g., machine learning), specialized models (e.g., neural networks), and clinical applications; and (3) Medical contexts targeted NO’s core therapeutic areas: cardiovascular, respiratory, and critical care medicine. Preliminary validation identified environmental science terms as major noise sources, which were subsequently excluded. The complete search syntax is shown in **Table 1**.

**Table 1:** Search strategy formula for NO-AI field in the Web of Science Core Collection database

#1	TS=("nitric oxide" OR "endothelium-derived relaxing factor" OR "EDRF" OR "nitrosative stress" OR "nitric oxide synthase" OR "NOS" OR "iNOS" OR "eNOS" OR "exhaled nitric oxide" OR "FeNO" OR "nitric oxide donor*")
#2	TS=("artificial intelligence" OR "AI" OR "machine learning" OR "deep learning" OR "neural network*" OR "predictive model*" OR "data mining" OR "natural language processing" OR "computer vision" OR "support vector machine" OR "random forest" OR "reinforcement learning" OR "clinical decision support" OR "automated diagnos*" OR "wearable device*" OR "image recognition" OR "time series analysis" OR "generative adversarial network")
#3	TS=("medical" OR "clinical" OR "therapeutic*" OR "diagnos*" OR "toxicity" OR "cardiovascular disease*" OR "respiratory disease*" OR "sepsis" OR "critical care" OR "immune response" OR "wound healing" OR "antimicrobial" OR "biomarker discover*" OR "patient monitor*" OR "precision medicine" OR "drug delivery" OR "pharmacokinetics")
#4	TS=("environment*" OR "pollution" OR "air quality")
#5	#1 AND #2 AND #3 NOT #4

AI: Artificial intelligence; NO: nitric oxide; TS: topic search.

#### Inclusion criteria

The dataset included publications from January 2005 to December 2024. This timeframe was selected to capture both the emergence and maturation of AI applications in healthcare. Eligible documents were restricted to original research articles and review articles published in English. To ensure data integrity, duplicate entries were rigorously removed using CiteSpace. The process implemented multi-dimensional deduplication based on digital object identifiers (DOIs), article titles, and author information.

#### Exclusion criteria

Non-English publications were excluded to maintain linguistic consistency and ensure broad accessibility of the analyzed literature. Document types such as editorials, commentaries, conference abstracts, letters, and book chapters were omitted to focus exclusively on original research and review articles with substantive scientific content. Publications outside the 2005–2024 timeframe were excluded to align with the study’s objective of capturing AI’s emergence and maturation in healthcare. Articles that lacked explicit integration of NO and AI were removed to preserve thematic relevance.

#### Data export

The final dataset (384 publications) was exported as full records (including abstracts, references, and affiliation data) in plain text format, and then converted to CSV file for downstream analysis.

### Analytical framework

#### Tool implementation

The bibliometric analysis leveraged three complementary computational tools to examine distinct dimensions of the dataset. CiteSpace was employed to generate co-occurrence networks of keywords and references, identify citation burst terms indicative of emerging trends, and visualize temporal shifts in research foci through time-zone mapping. VOSviewer was utilized to construct and analyze collaborative networks among countries, institutions, and authors, quantifying partnership strength via co-authorship and co-citation metrics. Finally, the Bibliometrix R package was applied to conduct temporal trend analysis, supplemented by thematic evolution maps and productivity metrics, thereby providing a longitudinal perspective on research output and thematic dynamics.

#### Analytical dimensions

Our multidimensional bibliometric framework examined four interconnected domains to unravel the NO-AI research landscape. Geospatial collaboration patterns were evaluated through productivity metrics and cooperation density analyses, mapping the contributions and partnership intensity among nations and institutions. Scholarly impact was assessed via author productivity indices and reference co-citation networks, identifying influential researchers and foundational literature. Knowledge dissemination dynamics were investigated by profiling high-impact journals and their thematic foci, revealing key platforms driving research propagation. Finally, conceptual evolution was traced using keyword co-occurrence clustering combined with timeline slicing, which delineated thematic shifts and emerging interdisciplinary intersections over the study period.

## Results

### Annual publication trends

**Figure 2:**
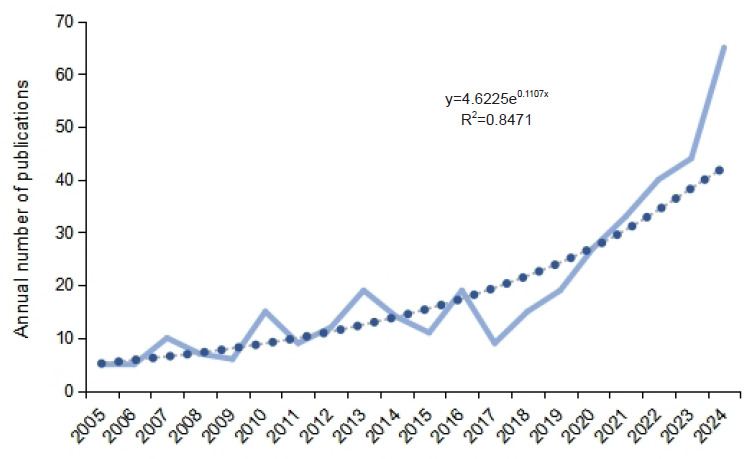
Annual publication trends in the artificial intelligence-driven nitric oxide field (2005–2024).

In recent years, research on the integration of NO and AI has gained increasing attention, with a gradual rise in the number of relevant publications. Analysis of annual publication trends from 2005 to 2024 reveals that research in this field has undergone two distinct phases (**Figure 2**).

During 2005–2016, the annual publication count showed a fluctuating growth, increasing from 5 publications in 2005 to 19 in 2016, indicating the nascent development stage in this field. The subsequent period (2017–2024) witnessed accelerated growth from an initial 9 publications in 2017 to a notable 65 publications by 2024, which is the highest recorded annual output in the study period. Trend fitting using a power-exponential model yielded a determination coefficient (*R*^2^ = 0.8471), confirming strong temporal consistency in publication growth and signaling the field’s transition into an active developmental phase.

### Analysis of country/institutional publications and collaboration networks

International research activities in the integration of NO and AI have shown an overall upward trend, with significant inter-country collaborative characteristics. As shown in **Table 2**, China ranked first globally with 119 publications, closely followed by the United States with 118 publications, indicating both countries’ dominant positions in publication output and research influence. England (29 publications), India (27), and Italy (27) occupied positions three through five. Canada ranked 6^th^ with 23 publications, followed by Australia, Japan, South Korea, and Germany, each contributing over 10 publications, demonstrating active global participation in NO-AI research.

**Table 2:** Top 10 countries in the artificial intelligence-driven nitric oxide field in the Web of Science Core Collection database

Rank	Country	Document	Total citation	H-index
1	China	119	1824	20
2	USA	118	4995	37
3	England	29	1445	18
4	India	27	454	12
5	Italy	27	1378	15
6	Canada	23	803	15
7	Australia	19	616	14
8	Japan	18	630	11
9	South Korea	16	448	6
10	Germany	14	599	9

**Figure 3:**
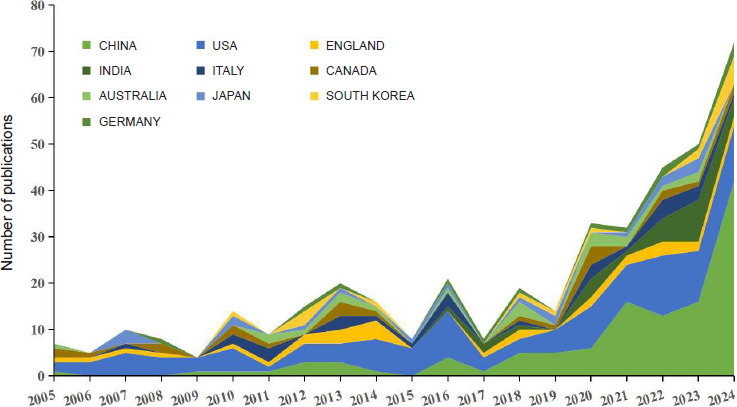
Annual publication trends of artificial intelligence-driven nitric oxide field in the top 10 contributing countries.

**Figure 3** depicts annual publication trends among the top 10 countries. China and the United States exhibited rapid growth in publication output after 2017, while England and Italy demonstrated slower annual growth rates. Although other countries maintain growth trends, research activity remained concentrated in the top-ranked nations.

**Figure 4:**
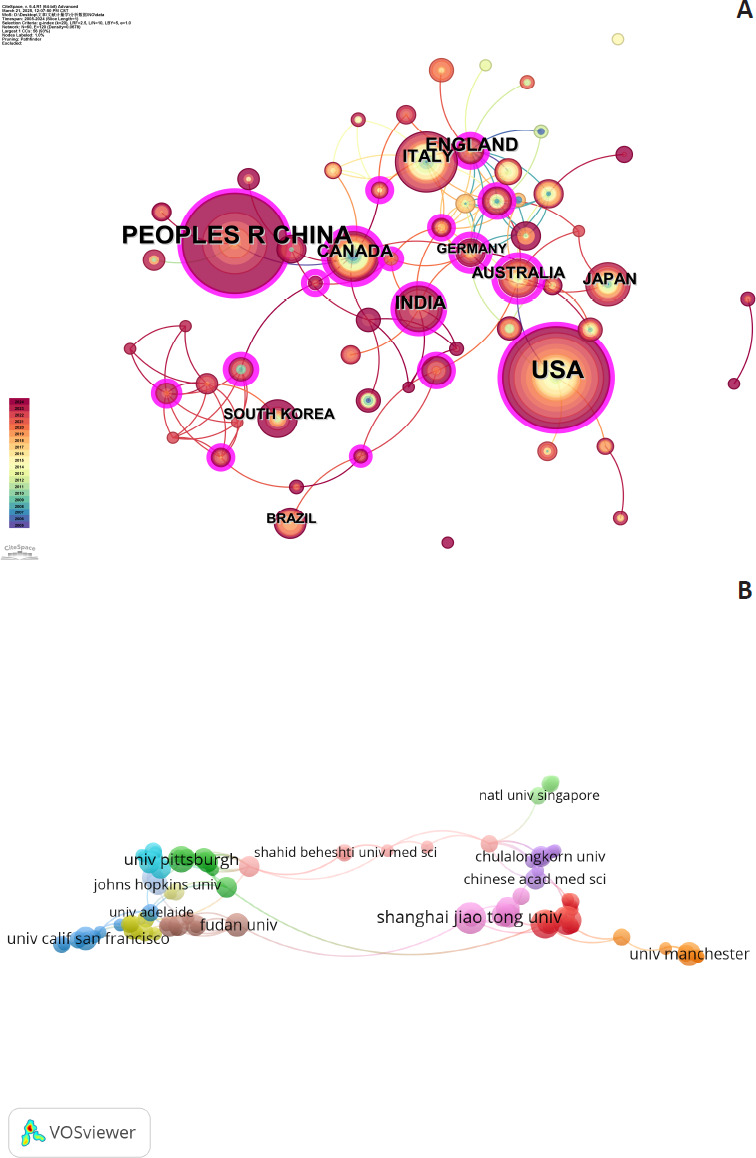
Country and institutional cooperation network of the artificial intelligence-driven nitric oxide field. (A) International collaboration network among countries. (B) Institutional collaboration network. (C) Global distribution of collaborations.

The country-level collaboration network generated by CiteSpace using a collaboration strength threshold > 10 (**Figure 4A**) revealed the most substantial international cooperation between China and the United States, with collaboration strength significantly surpassing other countries. Secondary collaborative relationships were observed between China and Australia, as well as China and Germany.

At the institutional level, leading research institutions in publication output are distributed across multiple countries, with the United States and China predominating. According to **Table 3**, the University of California System (United States) ranked first with 13 publications, demonstrating its leading research status in this field. Following closely were the Egypt Knowledge Bank (Egypt), Nanjing Medical University (China), and Shanghai Jiao Tong University (China), each with 8 publications, forming the second tier. Additionally, the Chinese Academy of Sciences, the Pennsylvania State System of Higher Education (United States), and Sichuan University (China) all contributed 7 publications, indicating significant research contributions from Chinese universities and research institutions.

**Table 3:** Top 10 institutions in the artificial intelligence-driven nitric oxide field in the Web of Science Core Collection database

Rank	Organization	Document	Total citations	H-index	Country
1	University of California System	13	359	9	USA
2	Egyptian Knowledge Bank	8	132	5	Egypt
3	Nanjing Medical University	8	62	4	China
4	Shanghai Jiao Tong University	8	60	5	China
5	Chinese Academy of Sciences	7	198	6	China
6	Pennsylvania Commonwealth	7	527	7	USA
	System of Higher Education				
7	Sichuan University	7	97	3	China
8	Chulalongkorn University	6	135	5	Thailand
9	Imperial College London	6	607	6	England
10	State University of New York	6	45	4	USA

The institutional collaboration network generated by VOSviewer (**Figure 4B**) further reveals collaboration patterns at the institutional level. The University of California System occupies a central position in the collaboration network, while Chinese universities and research institutions have formed relatively dense domestic collaborative groups. Furthermore, collaborations between differently colored clusters demonstrate the vitality of transnational cooperation.

### Literature and author collaboration networks

The distribution of highly cited articles in NO-AI clinical application studies reveals core research themes and pivotal contributions. As shown in **Table 4**, among the top 10 most cited works,^18^,^19^,^20^,^21^,^22^,^23^,^24^,^25^,^26^,^27^ Yuan et al.^18^ published in *ACS Nano* ranked first with 528 citations, indicating its substantial impact within the field. The second-ranked article, Kim et al.^19^ received 335 citations and focused on clinical risk assessment of related diseases. The third-ranked publication, Zannis et al.^20^ garnered 308 citations and addressed high-density lipoprotein biosynthesis.

**Table 4:** Top 10 highly cited publications in the artificial intelligence-driven nitric oxide field

Rank	Author	Publication year	Title	Journal	Journal IF (2023)	Total citations
1	Yuan et al.^18^	2020	Near-infrared light-triggered nitric-oxide-enhanced photodynamic therapy and low-temperature photothermal therapy for biofilm elimination	*ACS Nano*	15.8	528
2	Kim et al.^19^	2018	Retinopathy of prematurity: a review of risk factors and their clinical significance	*Survey of Ophthalmology*	5.2	335
3	Zannis et al.^20^	2006	Role of ApoA-I, ABCA1, LCAT, and SR-BI in the biogenesis of HDL	*Journal of Molecular Medicine*	4.8	308
4	Wilson and Baietto^21^	2011	Advances in electronic-nose technologies developed for biomedical applications	*Sensors*	3.4	277
5	Tomiyama and Yamashina^22^	2010	Non-invasive vascular function tests: their pathophysiological background and clinical application	*Circulation Journal*	3.1	254
6	McKhann et al.^23^	2006	Stroke and encephalopathy after cardiac surgery-an update	*Stroke*	7.9	244
7	Ferrini and De Koninck^24^	2013	Microglia control neuronal network excitability via BDNF signalling	*Neural Plasticity*	3	219
8	Wu et al.^25^	2014	Unsupervised phenotyping of Severe Asthma Research Program participants using expanded lung data	*Journal of Allergy and Clinical Immunology*	11.4	210
9	Endres et al.^26^	2008	Improving outcome after stroke: overcoming the translational roadblock	*Cerebrovascular Diseases*	2.2	197
10	Di Pietro et al.^27^	2016	Physiology and pathophysiology of oxLDL uptake by vascular wall cells in atherosclerosis	*Vascular Pharmacology*	3.5	181

IF: Impact factor.

**Figure 5:**
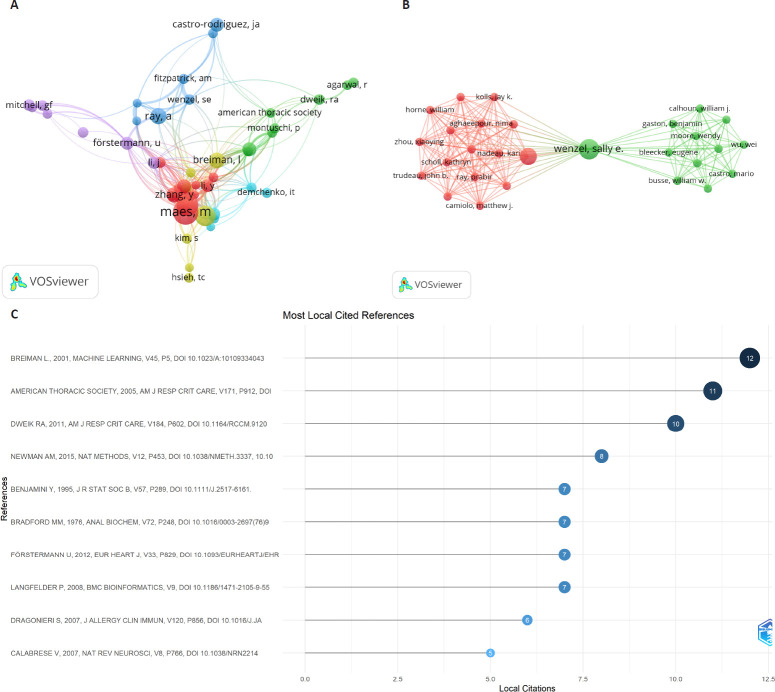
Author collaboration networks and literature rankings of the artificial intelligence-driven nitric oxide field. (A) Cited author collaboration network. Nodes represent individual authors, node size corresponds to citation frequency, and link thickness indicates collaboration intensity. (B) Author collaboration network. Node size represents the proportional to publication output, and link thickness represents collaboration frequency. (C) Top 10 most-cited external publications referenced in the artificial intelligence-driven nitric oxide literature corpus.

The highly cited literature distribution map generated by the Bibliometrix R package (**Figure 5A**) further clarifies citation patterns of seminal works. The analysis revealed Breiman^28^ as the most locally cited reference (12 local citations), followed by American Thoracic Society and European Respiratory Society^29^ (11 local citations) and Dweik et al.^30^ (10 local citations). These works constitute foundational references frequently cited by researchers in this domain.

Author-level analysis revealed that multiple academic teams have played pivotal roles in NO-AI integrated research. **Figure 5B** displays the collaboration network of cited authors. Breiman^28^ occupied the central hub of the network, maintaining strong collaborative ties with multiple researchers. Additionally, distinct collaborative clusters emerged, including a prominent group led by Maes,^31^,^32^ and Fitzpatrick et al.^33^ whose nodes occupy significant positions within the network, reflecting their broad engagement in interdisciplinary studies.

**Figure 5C** illustrates direct author collaboration networks. Two studies occupied a central position, forming intricate collaborative relationships with multiple researchers, resulting in a densely connected network.^34^,^35^ Secondary collaborative subgroups are also evident, indicating concentrated collaboration among core authors and distributed secondary team networks within the field.

### Core journals and cross-disciplinary collaboration network

The journal distribution pattern in NO-AI integrated clinical research demonstrates concentration and high-quality characteristics. As shown in **Table 5**, *ACS Nano* ranked first with 526 citations, highlighting its prominent academic standing in the field. *Survey of Ophthalmology* followed with 335 citations, focusing on ophthalmology-related research. *Journal of Allergy and Clinical Immunology* and *Journal of Molecular Medicine* ranked third and fourth with 311 and 308 citations, respectively.

**Table 5:** Top 10 core journals in the artificial intelligence-driven nitric oxide field by citation frequency

Rank	Journal	Total citations	IF (2023)	JCR (2023)	Founding year
1	*Acs Nano*	526	2.9	Q1	2020
2	*Survey of Ophthalmology*	335	5.2	Q1	2018
3	*Journal of Allergy and Clinical Immunology*	311	11.4	Q1	2014
4	*Journal of Molecular Medicine*	308	4.8	Q1	2006
5	*Sensors*	296	3.4	Q2	2011
6	*Circulation Journal*	254	3.1	Q2	2010
7	*Stroke*	252	7.9	Q1	2006
8	*Neural Plasticity*	244	3	Q2	2013
9	*Chest*	240	9.5	Q1	2010
10	*Cerebrovascular Diseases*	210	2.2	Q3	2008

IF: Impact factor; JCR: Journal Citation Reports.

Most top-ranked journals belong to Q1 or Q2 categories, maintaining relatively high impact factors. Notably, *Journal of Allergy and Clinical Immunology* achieved an impact factor of 11.4, reflecting its authoritative status in medical research.

**Figure 6:**
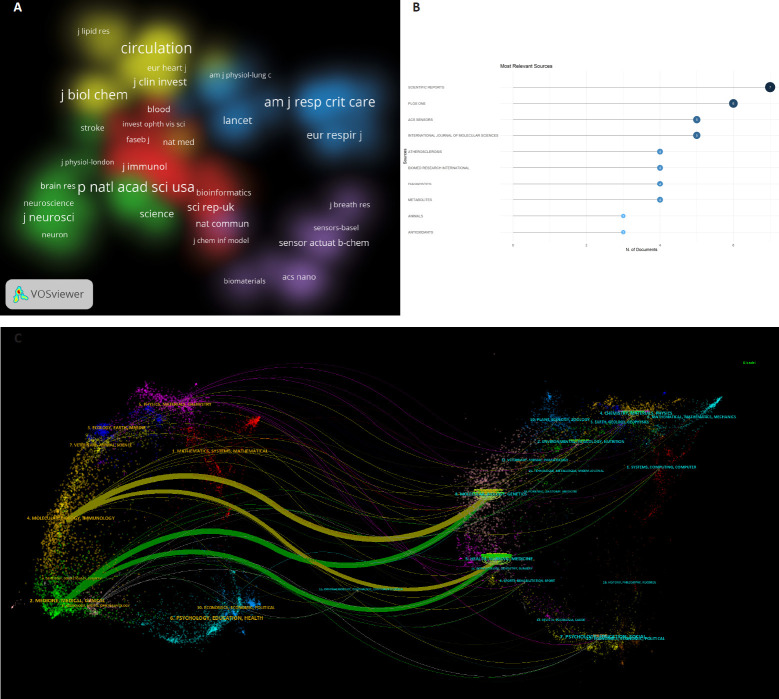
Core journals and cross-disciplinary collaboration network of the artificial intelligence-driven nitric oxide field. (A) Journal density distribution map. (B) Top 10 most productive journals. (C) The dual-map overlay of journals.

**Figure 6A**, generated using VOSviewer, presents a journal density map that further elucidates the distribution of high-density journals. The density visualization reveals core journals such as *Circulation* and *Journal of Biological Chemistry* occupying central positions in research hotspots, demonstrating a centralized yet diversified distribution across disciplines including molecular biology, medical chemistry, and fundamental medicine.

**Figure 6B** indicates that among the top 10 journals by publication output, *Scientific Reports* and *PLoS One* exhibit substantial contributions to this research domain, ranking first and second, respectively. **Figure 6C**, a dual-journal overlay map generated via CiteSpace, provides a clearer visualization of the distribution of citing journals and cited journals along with disciplinary fluidity. The result showed citing journals predominantly cluster in health sciences, clinical medicine, and biomedical fields, while cited journals are principally distributed across molecular biology and genetics-related disciplines. This disciplinary mobility confirms the interdisciplinary nature of NO-AI integrated research.

**Figure 7:**
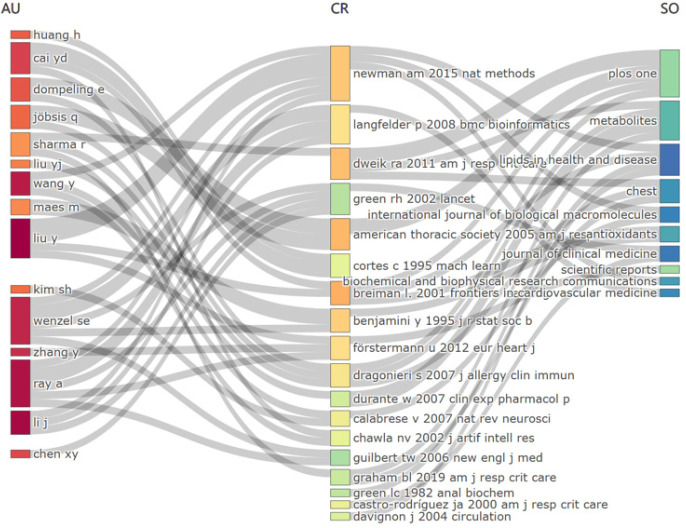
The tripartite network of author–literature–journal in the artificial intelligence-driven nitric oxide field. AU: Author; CR: reference; SO: source.

**Figure 7** illustrates the tripartite network structure integrating authors, literature, and journals, clearly revealing complex interconnections among these entities. Within the author network, core researchers including Huang H and Cai YD demonstrate highly cited publications, showing strong associations with influential works such as Newman et al.^36^ and Langfelder and Horvath.^37^ These works are predominantly published in high-impact journals including *ACS Nano*, *Scientific Reports*, and *Journal of Biological Chemistry*. The tripartite network structure further demonstrates that core research teams represented by Wenzel SE, Maes M, and Liu Y have established extensive collaborative relationships with both highly cited literature and authoritative journals across multiple disciplines. This tightly interwoven cross-domain collaboration pattern strengthens the research network in NO-AI convergence studies and drives its sustained development.

### Keyword evolution and research hotspot trends

**Figure 8:**
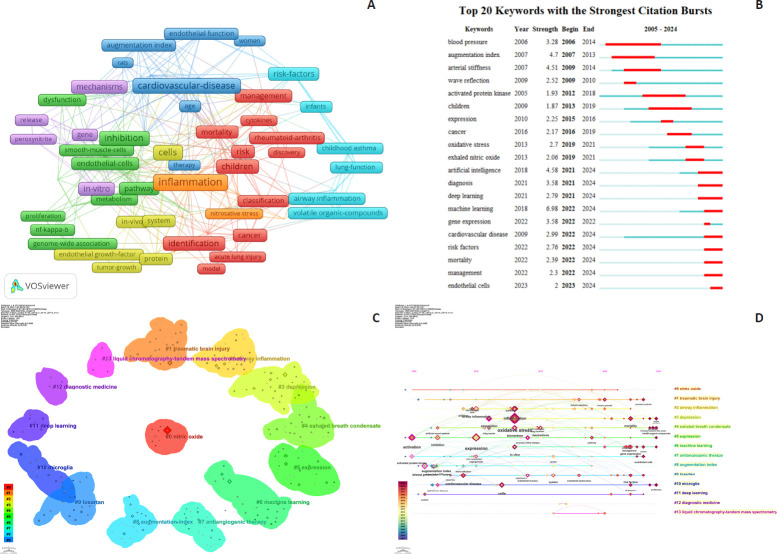
Evolutionary logic of keyword hotspots of the artificial intelligence-driven nitric oxide field. (A) Keyword co-occurrence network. (B) Top 20 burst keywords. (C) Keyword cluster map. The distinct color-coded clusters represent thematic groupings. (D) Timeline of the 13 largest keyword clusters.

The distribution of keywords in this study reveals disciplinary priorities and critical research themes. As shown in the keyword co-occurrence network (**Figure 8A**), core terms such as “inflammation,” “cardiovascular disease,” “nitric oxide,” and “machine learning” occupy central network positions, forming strong connections with emerging keywords like “deep learning” and “oxidative stress.” These associations reflect multidisciplinary integration and core scientific challenges, such as the application of machine learning in cardiovascular inflammation research.

Keyword burst detection analysis identifies temporal patterns of research focus (**Figure 8B**). For instance, “blood pressure” emerged as an early hotspot (2006–2014), while “machine learning” and “deep learning” demonstrated significant bursts from 2016–2024, indicating rapid technological advancements. The term “exhaled nitric oxide” exhibited a burst period (2012–2019), highlighting its potential in AI-driven precision medicine applications.

The keyword clustering map provides logical categorizations (**Figure 8C**). Identified clusters include “#0 diagnostic medicine,” “#1 exhaled nitric oxide,” and “#2 microbiome,” demonstrating thematic concentrations across research domains. Technical applications in NO quantification are represented by clusters such as “mass spectrometry” and “liquid chromatography tandem mass spectrometry.” The keyword timeline illustrates temporal evolution (**Figure 8D**), with “nitric oxide” persisting throughout the timeline, while “deep learning” and “diagnostic medicine” emerged as dominant post-2016 research foci.

**Figure 9:**
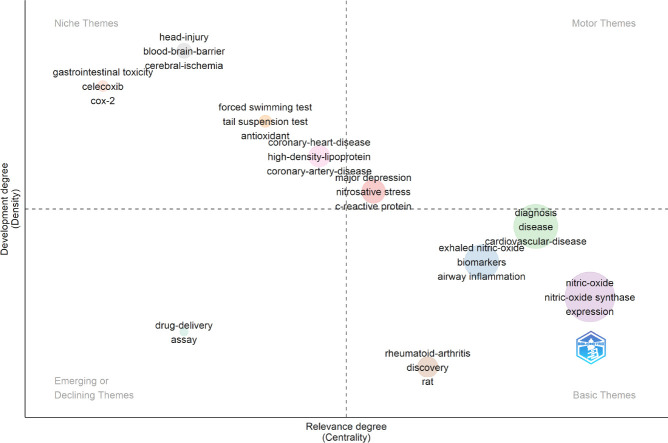
Strategic theme map in the artificial intelligence-driven nitric oxide field.

The research hotspot evolution map elucidates dynamic shifts in disciplinary themes and core developmental domains. The hotspot distribution map categorizes keywords into four quadrants (**Figure 9**): (1) Basic themes quadrant: High-centrality and low-density keywords dominate, including “nitric oxide,” “nitric oxide synthase,” “diagnosis,” “exhaled nitric oxide,” “disease,” and “rheumatoid arthritis,” primarily focused on precise diagnosis and treatment of disease characteristics and pathological processes. (2) Motor themes quadrant: Contains high-density but high-centrality keywords such as “augmentation index,” “endothelial function,” and “coronary heart disease,” addressing cardiovascular functional assessment and disease progression. (3) Niche themes quadrant: Features low-centrality yet high-density keywords like “head injury,” “blood-brain barrier,” “cerebral ischemia,” “celecoxib,” and “COX-2,” highlighting cerebral injury mechanisms and pharmacological interventions. (4) Emerging/declining themes quadrant: Comprises low-centrality and density keywords (e.g., “drug delivery” and “assay”), encompassing NO detection technologies and drug delivery systems. These quadrants collectively characterize the distribution characteristics of different research hotspots.

**Figure 10:**
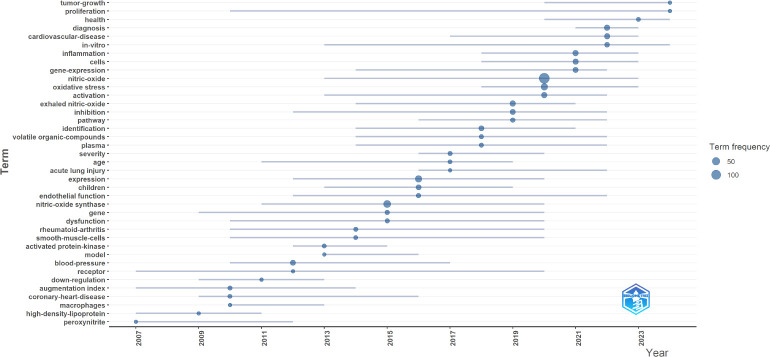
Theme trend keywords in the artificial intelligence-driven nitric oxide field.

**Figure 10** further reveals temporal trends in research foci. The timeline demonstrates a gradual transition from traditional themes like “inflammation” to emerging directions such as “machine learning” and “deep learning,” forming an evolutionary trajectory from classical biomedical research to AI-enabled applications. Notably, persistent clinical relevance is observed for themes like “cardiovascular disease” and “oxidative stress.”

## Discussion

### Discipline dynamics and driving forces of publication trends

From 2005 to 2024, interdisciplinary research on NO-AI exhibited a distinct two-phase growth pattern, with accelerated growth after 2017 aligning with global breakthroughs in medical AI. This trend is attributed to advancements in deep learning (e.g., algorithmic innovations driven by AlphaGo in 2016) and the demand for deciphering NO’s complex metabolic networks. Compared to AI’s early integration in oncology, NO-AI research demonstrated delayed initiation but notable growth momentum (24.5% compound annual growth rate 2017–2024), highlighting its technological potential. However, in contrast with AI’s 80% clinical translation rate in imaging diagnostics, NO-AI applications remain predominantly laboratory-validated, likely due to challenges such as high-noise NO monitoring data (e.g., environmentally influenced exhaled NO measurements) and prolonged clinical validation cycles (e.g., 3–5 years for multicenter trials of inhaled NO therapies). Future efforts should adopt a “data–algorithm–clinical” closed-loop framework in the field of oncology to accelerate technological iteration.

### Research ecosystem of country and institutional collaboration

China and the United States dominate with 237 combined publications, reflecting their strategic prioritization of medical AI and resource concentration. Exemplary contributions from institutions like the University of California System and Nanjing Medical University highlight the efficiency of “university-hospital” synergy. However, interregional collaborations exhibit a core-periphery structure, with Sino-American partnerships disproportionately overshadowing other combinations, potentially fostering research homogeneity.

### Knowledge flow patterns in literature-author networks

Highly cited works focus on photodynamic therapy (e.g., Yuan et al.^18^) and disease risk assessment (e.g., Kim et al.^19^), revealing dual “technology-driven” and “clinically-oriented” pathways. Notably, only 2 of the top 10 cited publications address AI model optimization, while the others emphasize the fundamental mechanisms of NO, suggesting that AI remains tool-dependent. Author networks further reveal a disconnect: core teams (e.g., Breiman L, and Wenzel SE) prioritize algorithmic development, whereas clinical expert engagement remains limited, causing model-clinical scenario misalignment. Comparatively, oncology AI achieves end-to-end optimization through “clinical-computational” deep collaboration.^38^ This field urgently requires interdisciplinary laboratories to bridge clinical needs and algorithmic design.

### Interdisciplinary attributes of core journals

High-impact journals demonstrate disciplinary fragmentation: material science journals (e.g., *ACS Nano*) emphasize NO delivery technologies, while clinical journals (e.g., *Journal of Allergy and Clinical Immunology*) focus on immune mechanisms. This fragmentation reflects interdisciplinarity but hinders systemic integration. For instance, *Scientific Reports* serves predominantly as a “fast-track” for technical validation, evidenced by its lower local citation rate *versus* specialty journals. The absence of computer science venues (e.g., NeurIPS) highlights algorithm-clinic publication barriers. We recommend adopting *Nature Biomedical Engineering*’s model by establishing NO-AI special sections with dual “computational-clinical” submission mechanisms.

### Evolutionary logic of keyword hotspots and clinical translation bottlenecks

This bibliometric analysis reveals the keyword hotspots and dynamic evolutionary characteristics in the NO-AI convergence field, providing critical insights into the core research directions and technological translation barriers.

Through co-occurrence, burst, and clustering analyses of keywords from 2005 to 2024, the following key trends and challenges emerge: During the early research phase (2005–2015), dominant keywords such as “blood pressure,” “inflammation,” and “nitric oxide synthase” reflected foundational investigations into NO’s biological mechanisms and cardiovascular roles, with studies focusing on physiological effects and clinical detection technologies (e.g., FeNO monitoring^10^,^11^), while AI applications remained nascent. For example, a 2010 study in *Circulation Journal* employed traditional statistical models to analyze NO-blood pressure regulation,^22^ with AI limited to auxiliary data classification. Post-2016, the emergence of keywords like “machine learning” and “deep learning” (burst strength > 5.0, persisting through 2024) marked a paradigm shift aligned with global AI advancements (e.g., generative adversarial networks, transformer models), driving research toward AI-optimized NO dosing, toxicity prediction, and smart monitoring systems. However, this technology-driven evolution exposed clinical algorithm misalignment: despite convolutional neural network-based FeNO prediction models achieving > 90% sensitivity in single-center studies,^19^,^39^ their real-world generalizability remains limited. In contrast, oncology’s integration of multi-omics data and clinical phenotypes^40^ has established closed-loop “data-algorithm-therapy” frameworks, whereas NO-AI research lacks systematic translation pathways.

Keyword cluster analysis revealed four major thematic groups: “diagnostic medicine,” “exhaled nitric oxide,” “microbiome,” and “oxidative stress.” The strong association between “diagnostic medicine” and “exhaled nitric oxide” underscores the central role of AI in non-invasive diagnosis of NO-related diseases (e.g., asthma, pulmonary hypertension). However, weak inter-cluster connections (“microbiome”–”oxidative stress” edge weight < 0.3) expose research gaps: while NO exerts dual regulatory effects on host-pathogen interactions in infectious diseases, AI applications in this domain remain virtually unexplored. In contrast, AI models in tumor immunotherapy have successfully deciphered dynamic networks of immune cells and cytokines within tumor microenvironments,^41^,^42^ whereas NO-AI research lacks comparable multi-scale analytical frameworks. Furthermore, isolated distribution of keywords like “reactive oxygen species” and “antioxidant” within the “oxidative stress” cluster suggests underutilized potential of AI in resolving NO-free radical equilibrium. Future efforts should adopt systems biology approaches to construct dynamic NO metabolic network models, integrating AI to predict regulatory mechanisms across pathological states.

Burst keyword analysis further delineates translational bottlenecks. For instance, “exhaled nitric oxide (FeNO)” as a 2012–2019 burst term saw declining prominence post-2020, directly correlated with the barriers to the clinical adoption of FeNO detection. Despite AI-enhanced FeNO data analysis efficiency, its primary care implementation remains constrained by equipment costs and operational complexity. Emerging burst terms like “generative adversarial networks (GANs)” (2021–2024) are currently restricted to NO drug design, neglecting their potential in dynamic dose optimization and toxicity simulation. Future research could leverage GANs to generate virtual patient cohorts for simulating long-term NO therapy effects, thereby compressing clinical trial timelines.

Current limitations primarily concern data quality and model interpretability. High-noise NO monitoring data (environmental interference, inter-individual metabolic variability) hinder multicenter standardization, yet paradoxically drive innovation in noise-robust AI architectures. Concurrently, black-box algorithms (e.g., deep learning) face clinical distrust due to interpretability deficits, though their superior pattern recognition capabilities remain indispensable for decoding nonlinear NO metabolic interactions. While explainable AI techniques (SHapley Additive exPlanations values, local interpretable model-agnostic explanations) are widely adopted in oncology prognostic models,^43^,^44^,^45^ their penetration into NO-AI research remains insufficient, highlighting an urgent need for domain-specific adaptation rather than mere technical transplantation. This dichotomy underscores the necessity for synergistic development of algorithmic transparency and clinical validity in NO-AI systems.

### Limitations and future directions

This study has three primary limitations: First, data sources were restricted to the Web of Science Core Collection, potentially excluding innovative findings from preprint platforms (e.g., arXiv) or regional databases (e.g., PubMed), which may introduce selection bias in technology trend identification. Second, despite optimized search strategies, rapid terminological evolution in AI (e.g., emerging technologies like Transformer models) may have led to incomplete lexical coverage. Third, the temporal scope of included literature concludes in 2024, failing to incorporate emerging studies published through April 2025, which may constrain the timeliness of trend extrapolation in this rapidly evolving field. Future research should focus on: (1) establishing multimodal NO dynamic metabolism databases to address data heterogeneity; (2) advancing explainable AI applications in toxicity early-warning systems while implementing terminological tracking mechanisms to accommodate rapid lexical evolution in AI domains; (3) designing prospective clinical trials for NO therapies using the FDA’s AI medical device approval framework to accelerate clinical implementation.

## Conclusion

This study systematically analyzes research trends in the NO-AI field (2005–2024) through bibliometrics, revealing its knowledge structure, collaboration networks, and developmental trajectories. We construct the first knowledge graph for the “NO-AI” interdisciplinary domain, addressing the gap in systematic analysis of cross-disciplinary research hotspots and evolutionary pathways. Key findings include: (1) Publication output exhibited a two-phase growth pattern, with accelerated expansion after 2017 coinciding with AI’s clinical penetration; (2) China and the United States dominated research productivity, yet international collaboration networks displayed a “core-periphery” structure, with a thematic focus on cardiovascular and respiratory diseases; (3) Highly cited works emphasized technology-driven innovations (e.g., photodynamic therapy), while deep integration of AI with NO’s fundamental mechanisms remains underdeveloped; (4) Keyword evolution reflects a paradigm shift from traditional mechanistic exploration to AI tool development, though clinical translation faces bottlenecks in data heterogeneity and algorithmic interpretability. These findings provide empirical evidence for understanding NO-AI research’s disciplinary positioning, translational barriers, and future directions.

NO-AI convergence research represents a technological innovation in medical gases and a paradigm case of AI-powered clinical transformation. Despite the current gap between technological development and clinical translation, standardized data protocols, interdisciplinary collaboration, and policy support could bridge the gap between “bench-side innovation” to “bed-side application.” This work not only offers a panoramic reference for researchers but also informs policymakers in optimizing resource allocation and translational strategies. As AI technologies evolve and multimodal data integration becomes more advanced, NO-AI research may emerge as a key driver in redefining therapeutic paradigms and reshaping clinical practice.

## Data Availability

*Not available*.
